# Monitoring the effects of dexamethasone treatment by MRI using *in vivo* iron oxide nanoparticle-labeled macrophages

**DOI:** 10.1186/ar4588

**Published:** 2014-06-23

**Authors:** Azza Gramoun, Lindsey A Crowe, Lionel Maurizi, Wolfgang Wirth, Frank Tobalem, Kerstin Grosdemange, Geraldine Coullerez, Felix Eckstein, Marije I Koenders, Wim B Van den Berg, Heinrich Hofmann, Jean-Paul Vallée

**Affiliations:** 1 Department of Radiology and Medical Informatics, Faculty of Medicine, Foundation for Medical Research, University of Geneva, 64 Avenue de la Roseraie, CH-1211 Geneva, Switzerland; 2 Institute of Anatomy, Paracelsus Medical University, Strubergasse 21, AT-5020 Salzburg, Austria; 3 Powder Technology Laboratory, Ecole Polytechnique Fédérale de Lausanne, Station 12, CH-1015 Lausanne, Switzerland; 4 Department of Rheumatology, Rheumatology Research and Advanced Therapeutics, Experimental Rheumatology, Radboud University Nijmegen Medical Centre, 272 Geert Grooteplein 26-28, PO Box 9101, 6500 HB Nijmegen, Netherlands

## Abstract

**Introduction:**

Rheumatoid arthritis (RA) is a chronic disease causing recurring inflammatory joint attacks. These attacks are characterized by macrophage infiltration contributing to joint destruction. Studies have shown that RA treatment efficacy is correlated to synovial macrophage number. The aim of this study was to experimentally validate the use of *in vivo* superparamagnetic iron oxide nanoparticle (SPION) labeled macrophages to evaluate RA treatment by MRI.

**Methods:**

The evolution of macrophages was monitored with and without dexamethasone (Dexa) treatment in rats. Two doses of 3 and 1 mg/kg Dexa were administered two and five days following induction of antigen induced arthritis. SPIONs (7 mg Fe/rat) were injected intravenously and the knees were imaged *in vivo* on days 6, 10 and 13. The MR images were scored for three parameters: SPION signal intensity, SPION distribution pattern and synovial oedema. Using 3D semi-automated software, the MR SPION signal was quantified. The efficacy of SPIONs and gadolinium chelate (Gd), an MR contrast agent, in illustrating treatment effects were compared. Those results were confirmed through histological measurements of number and area of macrophages and nanoparticle clusters using CD68 immunostaining and Prussian blue staining respectively.

**Results:**

Results show that the pattern and the intensity of SPION-labeled macrophages on MRI were altered by Dexa treatment. While the Dexa group had a uniform elliptical line surrounding an oedema pocket, the untreated group showed a diffused SPION distribution on day 6 post-induction. Dexa reduced the intensity of SPION signal 50-60% on days 10 and 13 compared to controls (*P* = 0.00008 and 0.002 respectively). Similar results were found when the signal was measured by the 3D tool. On day 13, the persisting low grade arthritis progression could not be demonstrated by Gd. Analysis of knee samples by Prussian blue and CD68 immunostaining confirmed *in vivo* SPION uptake by macrophages. Furthermore, CD68 immunostaining revealed that Dexa treatment significantly decreased the area and number of synovial macrophages. Prussian blue quantification corresponded to the macrophage measurements and both were in agreement with the MRI findings.

**Conclusions:**

We have demonstrated the feasibility of MRI tracking of *in vivo* SPION-labeled macrophages to assess RA treatment effects.

## Introduction

Rheumatoid arthritis (RA) is one of the most common inflammatory bone conditions with a rising prevalence due to the increase in the life expectancy of the population. Because of its chronic nature and bone destructive effects, RA is associated with a high rate of disability and morbidity among patients, substantially diminishing their quality of life [1]. Currently, diagnosis of RA mainly depends on clinical scoring, serum rheumatoid factor and identification of erosive lesions on conventional radiographs. These methods do not allow early intervention at a point when permanent joint damage can be prevented. They also do not address the complicated nature of the disease where patients can show little or no signs of inflammation during ongoing episodes of joint damage [2]. Thus new technology allowing early onset and precise assessment and follow-up during and after treatment would be of great value to RA management.

Magnetic resonance imaging (MRI) is an advanced and sophisticated technology that is constantly evolving and can be used for imaging inflammatory bone diseases [3]. It is possible to detect joint effusion and synovial inflammation as well as bone and cartilage changes when non-contrast enhancement MRI is used. This is due to the fact that tissues show natural contrast due to their relative T1 (longitudinal) and T2 (transverse) relaxation properties, as protons in water and tissue are diamagnetic and therefore have some effect on the local magnetic field. However, contrast can be enhanced by the addition of agents that speed up these relaxation processes or disturb the local field. Traditional contrast agents such as gadolinium chelate (Gd), which is paramagnetic, weakly attract the local field causing mainly a reduction in T1 relaxation time by interaction of the tissue protons with its unpaired electrons. Therefore, a faster return to equilibrium of nearby protons takes place causing a hyperintense signal on T1-weighted images. The magnetic enhancement effects of Gd are amplified during inflammatory arthritis where the increase in vascular permeability induced by synovial inflammation allows a deeper diffusion into the tissues. While the use of conventional contrast agent has some benefits over non-enhanced imaging, it remains non-specific and does not provide cellular and molecular information. Thus, a more specific synovial contrast than that offered by non-enhanced and Gd-enhanced MRI would significantly improve RA diagnosis.

Nanotechnology is a rapidly growing field with a wide variety of applications, particularly in biomedicine. Among those applications are magnetic resonance (MR) diagnostic enhancement and molecular imaging using superparamagnetic iron oxide nanoparticles (SPION). These SPION applications exploit the strong paramagnetic effect of the nanoparticles in the presence of an external magnetic field due to their iron oxide nanocrystal core [4]. Several classifications of SPIONs exist that are based on the hydrodynamic particle size, polymeric coating material and their biomedical application. The classification most widely used is that based on the hydrodynamic particle size. Iron oxide nanoparticles with a mean hydrodynamic diameter that is more than 50 nm are those most widely used in clinical applications and are referred to as SPIONs, whereas those with a hydrodynamic diameter of less than 50 nm are known as ultra small superparamagnetic iron oxide (USPIO) particles [5].

SPIONs are considered to be T2 contrast agents interacting strongly with the local field causing a dephasing effect and loss of signal in that field. This is accompanied by a faster T2 relaxation time and causes the absence of signal from surrounding protons soon after the excitation pulse. Thus, the SPION MR signal is hypointense on both T1- and T2-weighted images. New sequences are now available that recover positive signal from such *negative* contrast agents by exploiting ultra-short echo times (signal acquired before the T2 decay) [6,7] or the off-resonance effects due to the disturbed local field [8,9].

In addition to their magnetic properties, the spontaneous phagocytosis of SPIONs by cells of the reticuloendothelial system [10] which are upregulated at the sites of infection and inflammation [11,12] have allowed researchers to track *in vivo* labeled cell populations in several pathological conditions. The dynamics of SPION phagocytosis control their biodistribution, which in turn depends on several physicochemical characteristics. Those characteristics include the size of the iron oxide crystals, the type of their polymeric coating, the hydrodynamic size of the coated particles and their charge. These parameters can be adjusted to modulate the plasma half-life of SPIONs and thus modulate their biodistribution and their uptake by monocytic cells, which in turn determines the biomedical applications of each type of SPION.

The feasibility of using SPION technology to enhance MR imaging of arthritis models is well documented [13-25]. However, most of these are proof-of-principle and validation studies of this tool to assess phagocytic activity at one particular time point. Consequently, they provide little or no information on the potential of the methods for follow-up studies. It is currently not known if SPION can be used for cell tracking during the progression of experimental arthritis nor the evaluation of RA treatments effects *in vivo*.

The goal of the current investigation was to evaluate *in vivo* SPION-labeled macrophages as a tool for determining RA treatment efficacy using MRI in antigen-induced arthritis (AIA). To that end, the AIA rat model was used and the effects of a glucocorticosteroid, dexamethasone (Dexa), on disease progression were compared to untreated arthritic animals during a period of two weeks. We determined the changes in signal from the synovium of the knee joint caused by SPION-labeled macrophages via qualitative and quantitative measurements. These MRI results were then verified using thorough histological measurements of both SPIONs and macrophages, confirming the accuracy of our MRI findings. Our data clearly demonstrate the validity of using SPION-labeled macrophages to experimentally assess RA treatments in a fast and non-invasive approach and suggest a possible clinical application for the evaluation of RA in patients.

## Methods

### Animals

Thirty-three female Lewis rats were obtained from Janvier Laboratories (Cedex, France). The rats weighed between 150 and 175 g and were 6 to 8 weeks old on arrival. They were housed in the animal facility at the University of Geneva under pathogen-free conditions in standard cages and were fed standard diet and water *ad libitum*. Animal handling was in accordance with guidelines of the Swiss Committee of Animal Experiments. The experimental protocol was approved by the Animal Care Committee at the University of Geneva (authorization number 1049/3580/3).

### Induction of antigen-induced arthritis

All experimental procedures, animal manipulation, and MR scanning were done under isoflurane inhalation anesthesia (Nicholas Piramal, London, UK) (1.5% O_2_ and air with 2 to 3% isoflurane). Animals were immunized by a subcutaneous injection of 120 μL emulsified solution of 500 μg methylated bovine serum albumin (mBSA) (Sigma Aldrich, St Louis, MO, USA)/50 μL saline plus 50 μL complete Freund’s adjuvant (CFA) (Difco Labs, Detroit, MI, USA) divided between four sites (two on the back between the shoulder joints and each front paw). The animals simultaneously received an intra-peritoneal injection of 2 × 10^9^ cells of heat-inactivated *Bordetella pertussis* (B.P.) (Lee Labs, Grayson, GA, USA) as an additional adjuvant. One week after the initial immunization, the animals received two booster subcutaneous injections (one near each hip joint) of 100 μL of the same mBSA/CFA emulsion and an additional intra-peritoneal B.P. injection was administered at the same concentration. Three weeks after the first immunization, mono-arthritis was induced in the right knee joint by an intra-articular injection of 500 μg mBSA in 50 μL saline, and the contra-lateral knee joint received 50 μL of saline and served as an internal control.

### Dexamethasone treatment protocol and contrast agents (SPION and gadolinium chelate) administration

For modulation of disease progression and macrophage behavior in the AIA model the following Dexa (Mephameson-4, Mepha Pharma, Basel, Switzerland) treatment regimen was used: 3 and 1 mg/kg Dexa was administered by intra-peritoneal injection on days 2 and 5 post-AIA induction respectively, and the controls (untreated group, with AIA) received an injection of the same volume of sterile saline via the same route.

Five h after the last Dexa injection, the animals received a single injection of 7 mg Fe/rat SPIONs intravenously in the tail vein. During each MR scanning session (starting 24 h after SPION injection) on day 6 and again on days 10 and 13 post-AIA induction 0.25 mmol Gd/rat (Gadoteric acid commercially available as Dotarem®, Guerbet, Cedex, France) was administered via an intravenous cannula inserted intra-peritoneally and fixed in place by a single suture to ensure identical animal position for the pre- and post-Gd MR images. Post-Gd MR images were obtained within 5 to 7 minutes after injection.

### SPION production and characterizing techniques

The naked SPIONs were manufactured by an aqueous co-precipitation method to obtain maghemite phase (γ-Fe_2_O_3_) [26,27]. Briefly, 0.064 moles of Fe^II^ from FeCl_2_ powder and 0.128 moles of Fe^III^ from FeCl_3_ powder (molar ratio Fe^II^:Fe^III^ = 1:2) were mixed in 1.5 L of water at room temperature with 120 ml of ammonia (NH_4_OH at 28%). After 10 minutes of reaction the suspension of magnetite (Fe_3_O_4_) was sedimented by applying a magnetic field gradient and washed three times with water until the pH reached 7. The naked SPION were then redispersed in 400 ml and oxidized with 160 ml of nitric acid (HNO_3_ at 2 M) and 240 ml of ferric nitrate (Fe(NO_3_)_3_ at 0.35 M) under reflux for 90 minutes. The black suspension became brown and was sedimented applying a magnetic field gradient and washed with water. The naked SPION of maghemite phase (γ-Fe_2_O_3_) was then dialyzed against HNO_3_ 10 mM for 3 days by changing the washing solution every 12 h. The suspension was finally centrifuged at 3 × 10^4^ G for 15 minutes and only the supernatant was kept.

Surface modification of the naked SPION with poly vinyl alcohol (PVA) was done following a protocol described previously [27-29]. Briefly, ten volumes of naked SPION were mixed with nine volumes of PVA (PVA-OH 3.85 with a molecular weight of 14,000 g/mol) solution at 100 mg_PVA-OH_/ml and one volume of vinyl alcohol/vinyl amine co-polymer (A-PVA M12 with a molecular weight of 80,000 to 140,000 g/mol) solution at 20 mg_A-PVA_/ml). The product will be referred to as SPIONs in this work.

Naked SPIONs and SPIONs iron concentrations (in mg_Fe_/ml) were measured with an induced coupled plasma atomic emission spectroscope (ICP-OES) after dissolution in hydrochloric acid solution (HCl at 6 M) and as such (without dissolution) using a magnetosusceptometer (MS3 from Bartington®, Oxon, England) [30]. Transmission electron microscopy micrographs of SPION samples were acquired using samples diluted to 50 μg_Fe_/ml to obtain the crystallite core sizes (referred to as cores size in Table 1). Number-weighted hydrodynamic diameters (referred to as particle size in Table 1) with polydispersity index (PDI in Table 1) of naked SPIONs and SPIONs were measured on photon correlation spectroscopy apparatus (PCS) at 400 μg_Fe_/ml. Zeta potentials of the two types of particles were also measured on the ZetaPals PCS apparatus at 400 μg_Fe_/ml at pH of approximately 7 adjusted with sodium hydroxide (NaOH at 0.1 M). The amount of PVA present on SPIONs was calculated after thermogravimetric analysis from 30 to 800°C (10°C/minute) under air (30 ml/minute). The saturation magnetization was obtained from freeze-dried powder of naked SPIONs using a superconducting quantum interference device (SQUID) [27].

**Table 1:** Naked SPION and SPION characterization

**Particles**	**Medium**	**Concentration, mg**_**Fe**_**/ml**	**Cores size, nm**	**Particle size (PDI), nm**	**Zeta potential at pH 7, mV**	**PVA/Fe ratio, mg**_**PVA**_**/mg**_**Fe**_	**Saturation magnetization, EMU/g**
Naked SPION	HNO_3_ 10 mM	10	7.2 ± 2.5	14 ± 2 (0.25)	0 ± 3	0	54
SPION	HNO_3_ 5 mM	5	7.2 ± 2.5	25 ± 3 (0.20)	+16 ± 3	9	54

SPION, superparamagnetic iron oxide nanoparticles; PDI, polydispersity index.

+/- are standard error for the core size and +/SD for the rest the parameters (Particle size and Zeta potential).

### MR imaging protocol

MR *in vivo* imaging of rat knee joints employed a Siemens Magnetom® Trio 3 T clinical scanner (Siemens AG, Erlangen, Germany) using the standard 4-cm loop coil and respiratory monitoring with a pressure pad. The contralateral left knee was imaged simultaneously as an internal control.

The protocol began with a standard low-resolution localization sequence and the isotropic resolution three-dimensional ultra-short echo time (UTE) double-echo MR sequence fixed orthogonal and at the magnet center [7,31]. These were subsequently used to localize the correct plane for the two-dimensional or thinner slab three-dimensional images as well as for quantitative analysis. The protocol took approximately 40 minutes and the parameters of the sequences used were as follows: (1) a three-dimensional T1-gradient echo (VIBE) was used to detect and visualize SPIONs by signal loss (negative contrast). Additionally, VIBE was also repeated after Gd administration through the intra-peritoneal cannula and without changing the position of the animal for visualization of synovial edema via non-specific contrast-agent uptake. The parameters were: reception time/echo time (TR/TE) 14.3/5.9 ms, flip angle 12°, fat suppression, isotropic resolution 0.31 mm, and field of view (FOV) 100 mm, acquisition time 4 minutes 54 seconds; (2) difference ultra-short echo-time imaging (dUTE) was used for detection and quantification of iron oxide particles positive contrast. SPION detection by the dUTE sequence consisted of the acquisition and subtraction of two echo times (ultrashort TE, and short TE(2)) leading to positive contrast from short T2* species and reduced signal elsewhere. The parameters were: three-dimensional isotropic matrix 448 and 80 mm FOV, giving 180 μm in all three dimensions, 50,000 radial projections, UTE/TE(2) 0.07 ms/2.46 ms (for in-phase fat/water image), TR 9.6 ms (*in vivo* 100 segments), flip angle 10°, acquisition time 16 minutes 54 seconds.

### Qualitative scoring of MR images

Three different parameters were defined and qualitatively scored to assess the evolution of AIA in the absence and presence of arthritis treatment on MR images. These parameters depended on the behavior of SPIONs and Gd in arthritic knees and included the following: the intensity and diffuseness of SPIONs in the inflamed synovium as well as the severity of synovial edema depicted by Gd infiltration. A scoring system from 0 to 3 was devised where a score of 0 denoted the lowest score and 3 the highest for any of these parameters (0 is absence of parameter). Blinded scoring was carried out by an experienced radiologist.

### SPION signal quantification using segmentation software and dUTE

dUTE provides quantifiable SPION signal related to concentration [32]. Three-dimensional volume segmentation exploiting the contrast and monotonic signal/concentration correlation of dUTE allows unknown heterogeneous regions of iron oxide uptake to be assessed in disease models after systemic injection (custom software, Paracelsus Medical University, Salzburg, Austria). The analysis software allows simultaneous segmentation of the two simultaneously acquired UTE and TE(2) images and the positive contrast iron oxide image. The semi-automatic threshold method selects regions of similar intensity with a radius constraint around a user-defined point and this is repeated for all the regions of SPIONs in three-dimensions. A histogram of pixel intensities in the dUTE image is obtained from which the mean intensity value and total volume in pixels and mm^3^ are reported.

### Histology sample preparation

Knee joints (both the femur and tibia) were isolated and fixed in 4% phosphate-buffered formalin for 48 h. Two embedding techniques were used to prepare the knee samples, which required two different methods of sample preparation: (1) for paraffin-embedded sections the samples were decalcified in 10% ethylenediaminetetraacetic acid (EDTA) (AppliChem, Darmstadt, Germany) for a period of 8 weeks. Samples were then embedded in paraffin and sectioned at 5 μm thickness; (2) For Technovit® 9100 New-embedded sections (Heraeus Kulzer, Hanau, Germany) the samples were dehydrated in increasing concentrations of ethanol for 24 h prior to xylene for 12 h at room temperature (RT). Samples were subjected to pre-infiltration before the infiltration solution (see manufacturer’s instructions) was added to the samples for a 48-h incubation period at 4°C. A freshly prepared polymerization solution was used to embed the knee samples that were then evacuated at 200 mbar for 5 minutes. The polymerization process took place between −2 and −20°C and was completed within 24 h. The embedded samples were cut using a special carbide blade at 5 μm thickness. Subsequently, the mounted sections were covered by parafilm and pressed overnight (ON) at 60°C.

### Histological staining

#### Immunohistochemistry

Paraffin sections were de-paraffinized, washed and blocked using peroxidase blocking buffer (Dako, Hamburg, Germany) before they were incubated with the primary antibody mouse rat CD68 antibody (ABD Serotec, Oxford, UK) at 1:50 titre for 1 h at RT in a moist chamber. After two washes, the slides were incubated with the secondary antibody, either peroxidase goat anti-mouse IgG (Dako) or fluorescein isothiocyanate (FITC) goat anti-mouse IgG (Jackson, West Grove, PA, USA) for 30 minutes at RT. After two washes, the slides were developed for 1 minute at RT using the one step AEC solution (Biogenex, Fremont, CA, USA). Slides were washed using washing buffer (Dako) twice as well as twice with water. Mayer’s hematoxylin (Sigma-Aldrich) was used as counterstaining. Technovit sections were deplastized using xylene, 2-methoxyethylacetate twice for 20 minutes each at RT followed by acetone twice for 5 minutes. Slides were blocked for 60 minutes using 5% normal horse serum (Linaris, Mannheim, Germany) and 1% BSA. Endogenous avidin and biotin activity were also blocked using avidin and biotin blocking solutions (Vector Lab, Burlingame, CA, USA) for 60 minutes each consecutively. The slides were washed in PBS before they were incubated with the primary antibody mouse anti-rat CD68 antibody (ABD) overnight (ON) at 1:100 titre in a 2% normal horse serum and 1% BSA solution. The following day, the slides were washed and endogenous perioxidase activity was blocked using 0.2% H_2_O_2_ for 15 minutes before adding the secondary antibody horse anti-mouse IgG (Linaris) for 60 minutes in 1% normal horse serum and 1% BSA. ABC solution (Vector) and DAB (Sigma-Aldrich) were used to develop the slides for 45 minutes and 5 minutes respectively. Finally, the slides were washed and counterstained using Mayer’s hematoxylin before mounting.

#### Prussian blue staining

Paraffin sections were de-paraffinized and rehydrated in water. A working solution of equal parts of 20% HCl and 10% K_4_Fe(CN)_6_ was incubated with the slides at RT for 20 minutes. After three washes with water, slides were counterstained using Nuclear Fast Red for 5 minutes. Finally, slides were washed, dehydrated and mounted. When performed following immunostaining, the working solution used was diluted to a final concentration of 1% HCl solution and 1% K_4_Fe(CN)_6_ and incubated with the slides for 3 minutes with no counterstaining.

Technovit sections were deplastized using xylene, 2-methoxyethylacetate twice for 20 minutes each at RT followed by acetone twice for 5 minutes. The slides were then pre-treated with 5% K_4_Fe(CN)_6_ for 5 minutes at 40°C. Subsequently, they were stained using 5% K_4_Fe(CN)_6_/HCl solution for 30 minutes at 40°C. After several washes, the sections were counterstained using Nuclear Fast Red, washed, dried and mounted.

### Quantification of immuno- and Prussian-blue staining

For image analysis, the number and percentage area of macrophages (CD68 immunostained cells depicted in brown) and SPIONs (Prussian-blue-stained particles depicted in blue) were quantified using three different types of software. Image J and Cell profiler were used to quantify different macrophage measurements and Definiens Tissue Studio 3® was used for SPION measurements. A semi-automated color selection and quantification process was used in the case of Image J and a fully automated batch processing script based on predetermined selection criteria was employed in case of Cell Profiler and Definiens Tissue Studio 3® (Definiens AG, München, Germany).

### Statistical analysis

Statistical evaluation was carried out using SPSS 21.0 for Mac using one-way analysis of variance (ANOVA) and the Dunnett T3 test. *P*-values <0.05 were considered statistically significant.

## Results

### SPION characterization

#### Core sizes: diameter of SPION measured by transmission electron microscopy (TEM) image analyses; particle size: hydrodynamic number-weighted diameter measured by PCS and given with polydispersity index

The physicochemical characteristics of naked SPIONs and SPIONs are summarized in Table 1. The naked SPIONs and SPIONs concentrations were 10 and 5 mg_Fe_/ml respectively. Crystallite core sizes measured by TEM were approximately 7 nm, which result in superparamagnetic properties confirmed by saturation magnetization of 54 emu/g and the absence of a hysteresis [33]. Moreover, the PVA coating did not affect either the SPION core size or the saturation magnetization. The hydrodynamic particle sizes corresponding to the aggregation state of the nanoparticles in aqueous suspension showed a difference between uncoated and coated SPIONs. When the SPIONs were naked, their average hydrodynamic size was approximately 14 nm and 25 nm with PVA coating. The increase of the hydrodynamic size was due to the adsorption of PVA on the particle surface. This measurement demonstrates the coating effect of PVA onto naked SPIONs. Zeta potential shifted from 0 to +16 mV due to PVA and amino-PVA coating providing a positive Zeta potential of SPIONs at physiological pH. Finally, thermogravimetric analysis confirmed the presence of PVA with the naked SPIONs in the mass ratio PVA/Fe equal to 9, as expected.

### MR imaging

**Figure 1:**
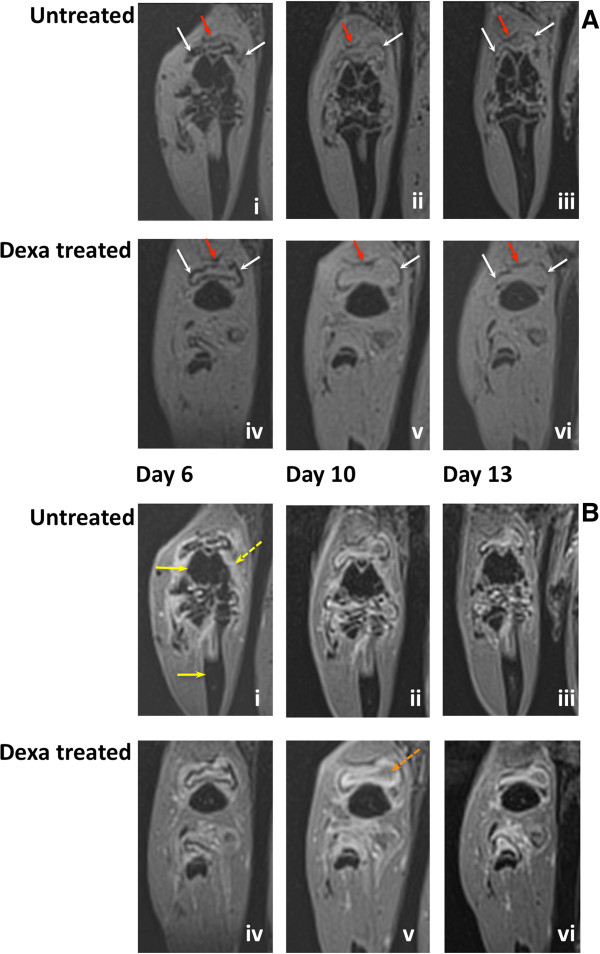
**The evolution of superparamagnetic iron oxide nanoparticles (SPION)-labeled macrophages signal on magnetic resonance (MR) images in the presence and absence of dexamethasone (Dexa).(A)** T1-weighted images (negative contrast) of SPION-labeled macrophages in the synovium during AIA. SPIONs were administered intravenously on day 5 post-AIA induction. Panel (i-iii) shows representative MR images from a control (untreated) animal with AIA and panel (iv-vi) shows MR images of a Dexa-treated animal on days 6, 10 and 13 post-AIA induction respectively. **(B)** T1-weighted images of SPION-labeled macrophages post-Gd administration of the same two animals shown in **A**. SPION signal is seen as a negative contrast, and Gd signal is seen as a positive contrast and depicts synovial edema. Panel (i-iii) shows representative MR images from a control (untreated) animal with AIA and panel (iv-vi) shows MR images of a Dexa-treated animal on days 6, 10 and 13 post-AIA induction respectively. White arrows, SPION signal (dark); red arrows, quadriceps tendon to patella (dark u-shaped line); yellow arrows: femur and tibia; broken yellow arrow, inflamed synovium (bright signal); broken orange line, edema pocket anterior to the femur. Similar results were seen in 13 untreated controls and 15 Dexa-treated animals. AIA, antigen-induced arthritis; Gd, gadolinium chelate.

To determine the validity of tracking *in vivo* SPION-labeled macrophages for the assessment of RA treatment effects and monitoring disease fluctuation, arthritic rats were treated twice with Dexa on days 2 and 5 post-AIA induction. A low dose of Dexa was chosen to modulate the course of arthritis without completely eradicating the underlying pathology. One dose of SPIONs (7 mg Fe/animal) was administered intravenously a few hours after the second dose of Dexa. *In vivo* MR imaging of the joints was conducted on days 6, 10 and 13 following AIA induction. In Figure 1, a representative series of MR images showing the distribution and evolution of the SPION-labeled macrophages in untreated and Dexa-treated groups can be seen. Two three-dimensional movies showing the detailed structures of the arthritic joint from the coronal and sagittal views with and without SPIONs can be seen in Additional files 1 and 2.

Images from the VIBE sequence depict the location of SPIONs, which cause a loss of MR signal and are seen as dark spots. In the upper panel A (i-iii), an inhomogeneous and diffused elliptical black line was found in the middle of a pocket of edema anterior to the femur and superior to the joint on day 6 (Figure 1A-i). This line gradually became more uniform and defined with time (Figure 1A ii and iii). The position of the line also became more peripheral in comparison to the edema and its intensity also decreased by day 13. This pattern was significantly different in the Dexa-treated group (Figure 1A iv-vi). Dexa-treated animals exhibited a distinct and uniform dark line as early as day 6, one day following SPION injection (Figure 1A-iv), and its intensity decreased quickly, almost disappearing by day 13 (Figure 1A v, vi). In comparison, VIBE post-Gd images of the same animals acquired a few minutes post-administration revealed a difference between the two groups on day 6 post AIA (Figure 1B i, iv) but no marked difference between the Dexa-treated and untreated groups on days 10 and 13 (Figure 1B ii, iii and v, vi). Gd highlighted synovial and extra articular edema (synovial inflammation) on VIBE, which otherwise could not have been visualized. The inability of Gd to demonstrate a difference between Dexa-treated animals and the control group was more evident at a later disease-stage where the Gd MR signal in the knee joint and adjacent tissues had decreased to a similar level in both groups (Figure 1B iii, vi). The rate of signal decrease was also comparable in both cases. This result was expected, as the extent of tissue infiltration and distribution of Gd is directly related to that of synovial inflammation, which resolves spontaneously with time in the AIA model (data not shown). For comparison, T1-weighted MR images without SPION administration, pre- and post-Gd on day 3, 6 and 10 are shown in Additional file 3.

### Qualitative assessment of MR images

**Figure 2:**
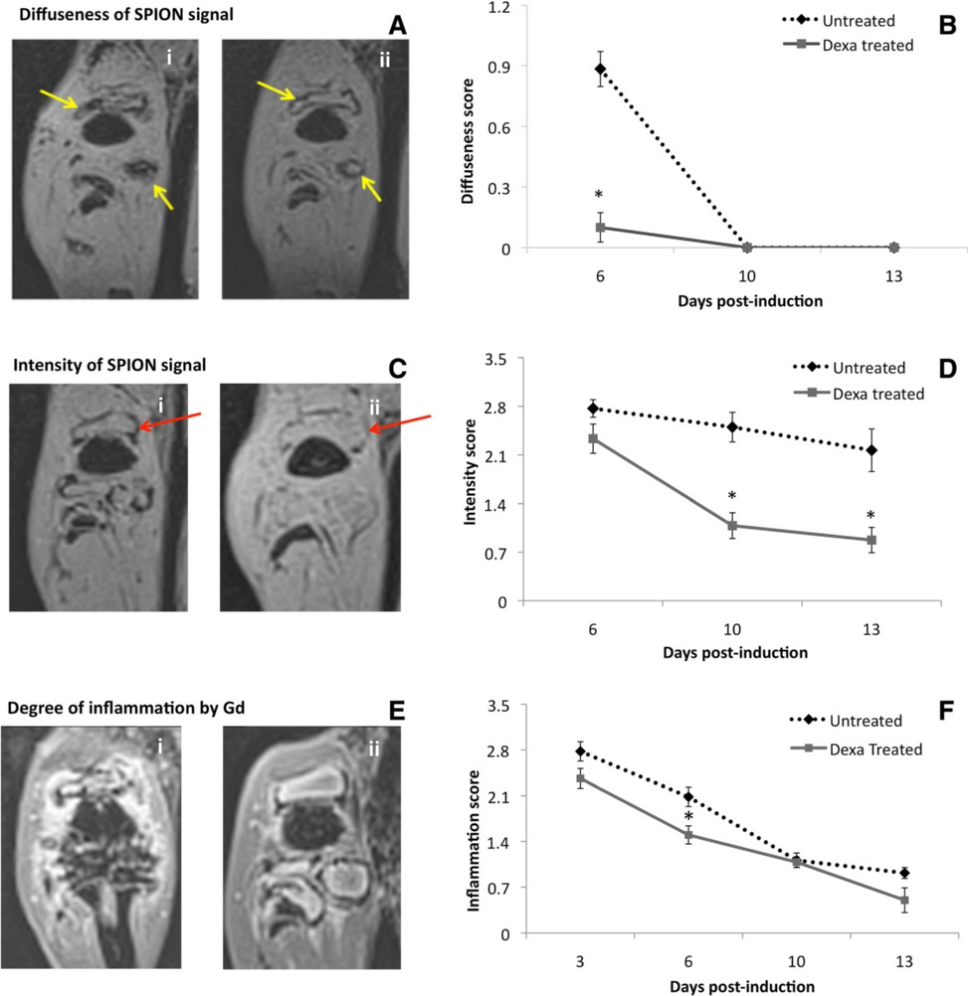
**Qualitative scoring of antigen-induced arthritis (AIA) stage using superparamagnetic iron oxide nanoparticles (SPION)-labeled macrophage and gadolinium chelate (Gd) signal in the synovium on days 6, 10 and 13 post-induction in the presence and absence of dexamethasone (Dexa). (A)** T1-weighted MR images showing the diffuseness of the SPION signal in the synovium; MR image with diffuseness (i) score of 3 and (ii) score of 0. **(B)** Line graph showing the blinded scores of diffuseness of the Dexa-treated and untreated groups. **(C)** T1-weighted MR images showing the intensity of the SPION signal in the synovium; MR image with intensity (i) score of 3 and (ii) score of 0. **(D)** Line graph showing the blinded scores of intensity parameter of the Dexa-treated and untreated groups. **(E)** Post-Gd T1-weighted MR images showing synovial inflammation as a positive contrast; MR image with synovial inflammation (i) score of 3 and (ii) score of 0. **(F)** Line graph showing the blinded scores of synovial inflammation of the Dexa-treated and untreated groups. All data points are mean ± standard error of mean. B and D: n = 13 untreated controls and n = 15 Dexa-treated group, reduced to 6 and 8 on day 13. B: P=1x10−8 on day 6, D: P = 0.00008 on day 10 and 0.002 on day 13 compared to the untreated control group. F: n = 20 on day 3, n = 14 on day 13, divided between Dexa-treated and untreated. P = 0.00126 on day 6 compared to the untreated controls. Red and yellow arrows indicate the SPION regions of interest where the scoring criteria were assessed. MR, magnetic resonance.

These findings were further corroborated via qualitative scoring of two criteria, diffuseness and intensity of SPION signal. Blinded scoring of diffuseness and intensity of SPION signal were conducted using a scale ranging from 0 to 3 on VIBE MR images. A score of 3 indicated high signal diffuseness and intensity (Figure 2A-i and C-i) and a score of 1 indicated low diffuseness and intensity (Figure 2A-ii and C-ii). A timeline of SPION signal plotted on days 6, 10 and 13 showed significantly high diffuseness of SPIONs in the untreated animals compared to their Dexa-treated counterparts, which scored very low in comparison (Figure 2B). By day 10, diffuseness scores of the untreated group dropped as low as the Dexa-treated group and remained unchanged up to day 13. In contrast, SPION intensity decreased more gradually over time yet remained elevated at all time points in the controls, whereas it decreased at a much higher rate with Dexa treatment and almost disappeared by day 13 (Figure 2D). This dissimilarity in SPION evolution resulted in a significant difference between the two groups on days 10 and 13 by which Dexa administration and efficacy could be clearly distinguished.Synovial inflammation was also assessed using a similar qualitative scoring system; however, signal intensity post-Gd administration on VIBE images was used in that case (Figure 2E i,ii). Although some effect of Dexa treatment on the volume of the synovial inflammation was detected on day 6, no difference between the two groups was found on days 10 and 13 (Figure 2F). In both cases, Gd demonstrated a gradual decrease in signal at a similar rate in the absence and presence of treatment. It was clear that Gd was influenced more by the disease state than by Dexa treatment. The Gd signal quantification was not only scaled to its signal intensity, but also to its regression reflected in the reduction in edema volume.

### Quantification of SPION MR signal

**Figure 3:**
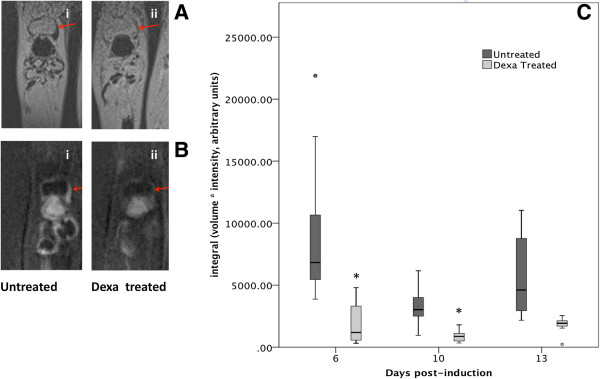
**Quantitative measurement of superparamagnetic iron oxide nanoparticles (SPION)-labeled macrophage signal in the presence and absence of dexamethasone (Dexa) using three-dimensional semi-automated segmentation software with difference ultra-short echo time (dUTE) magnetic resonance (MR) images. (A)** T1-weighted MR images of SPION-labeled macrophages in the synovium with varying hypointense signals (i) high (ii) low. **(B)** Corresponding dUTE MR images of the T1-weighted images seen in **A** with varying hyperintense (positive contrast) signal in the synovium (i) high (ii) low, that were used for three-dimensional signal segmentation. **(C)** Box plot graph comparing the SPION signal between the untreated and Dexa-treated groups using dUTE images. Data points ± standard error of the mean; n = 23, day 6; n = 22, day 10; n = 14, day 13. *P* <0.05 compared to the untreated control group (day 6, *P* = 0.00035; day 10, *P* = 0.00004; day 13, *P* = 0.0033).

The validation of the SPION intensity results was performed using semi-automated software for three-dimensional quantification of signal intensity. The measurements were conducted on dUTE images, which provided a positive SPION signal and a higher contrast with surrounding tissues allowing for easier and more precise signal segmentation (Figure 3A,B) and were compared between the groups (Figure 3). The signal intensity measurements were also highly accurate as they were based on both the three-dimensional volume of MR signal and the pixel intensity on every slice. Using this method, a difference between the Dexa-treated and untreated groups could be seen at all time points (Figure 3C).

### Histochemical and immunohistochemical staining analysis and quantification

**Figure 4:**
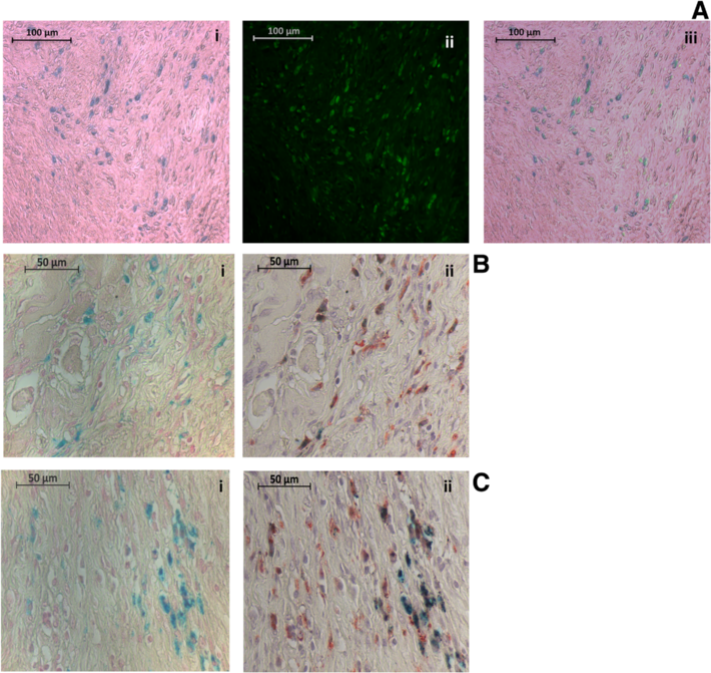
**Photomicrographs of Prussian blue and CD68-immunostained sections confirming *in vivo *superparamagnetic iron oxide nanoparticles(SPION) labeling of macrophages.(A)** Double-stained photomicrographs of (i) Prussian-blue-stained SPIONs (ii) immunofluorescent-stained macrophages using mouse anti-rat CD68 1^ry^ antibody (as a marker of macrophages) and anti-mouse fluorescein isothiocyanate (FITC) second antibody at 20 times magnification (iii) the overlay of the two previous images. **(B, C)** Photomicrographs of two separate examples of (i) Prussian blue followed by (ii) CD68-immunostained sections at 40 times magnification. Sections were stained consecutively and micrographs of the same region of interest were obtained.

**Figure 5:**
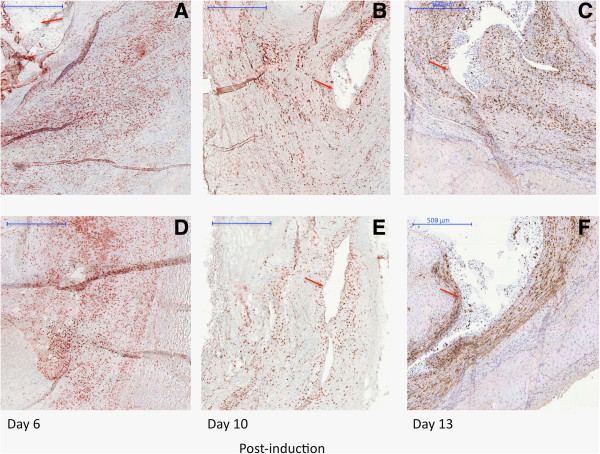
**Photomicrographs of CD68-immunostained sections comparing the evolution of macrophages during antigen-induced arthritis (AIA) in the presence and absence of dexamethasone (Dexa) treatment.** Photomicrographs showing macrophages (20 times magnification) on sections stained with a mouse anti-rat CD68 1^ry^ antibody and a peroxidase goat anti-mouse IgG second antibody. The slides were developed using one step AEC solution. Mayer’s hematoxylin was used as a counter staining. **(A, B, C)** Photomicrographs from untreated animals (control group) on days 6, 10 and 13 post-AIA induction. **(D, E, F)** Photomicrographs from Dexa-treated animals on days 6, 10 and 13 post-AIA induction. Red arrows indicate the edema pocket in the knee joint surrounded by inflamed synovial membrane with dense macrophage infiltration.

**Figure 6:**
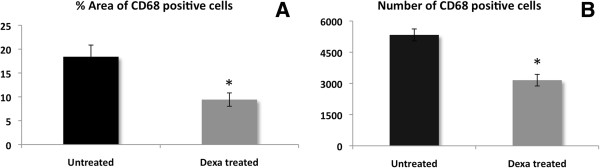
**Quantification of the area and number of CD68-positive macrophages on day 13 post-antigen-induced arthritis (AIA) induction.** Photomicrographs of CD68-immunostained sections on day 13 post-AIA induction were scanned and the images were analyzed for the % area of CD68-positive cells **(A)** and their number **(B)** using Image J and Tissue Studio® software, respectively. Four sections were quantified and averaged per animal. Data points are mean ± standard error of the mean and n = 4 per group. *P = 0.023 **(A)** and 0.003 **(B)** compared to the untreated control group.

**Figure 7:**
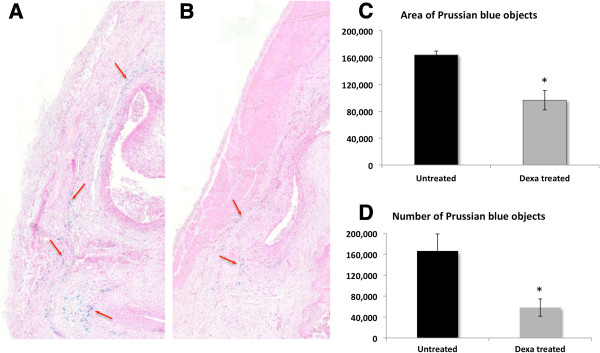
**Distribution and quantification of Prussian blue stained superparamagnetic iron oxide nanoparticles (SPIONs) on day 13 post-antigen-induced arthritis (AIA) induction.** Photomicrographs of Prussian-blue-stained sections showing an example of the distribution of SPIONs (red arrows) in the synovium of untreated animal **(A)** versus a Dexa-treated animal **(B)** on day 13 post-AIA induction at 1.5 times magnification. Quantification of the area **(C)** and number **(D)** of Prussian-blue-stained SPIONs on day 13 post-AIA induction. Photomicrographs of Prussian-blue-stained sections were scanned and the images were analyzed for the area **(C)** and the number **(D)** of SPIONs using Tissue Studio® software. Four sections were quantified and averaged per animal. Data points are mean ± standard error of the mean and n = 5 per group. *P = 0.005 **(A)** and 0.016 **(B)** compared to the untreated control group.

The MRI data were verified using histochemical and immunohistochemical staining of the knee joint. First, *in vivo* SPION uptake by macrophages and their distribution in the knee joint were assessed. To this end, Prussian blue staining was used to visualize SPION clusters and CD68 immunostaining was used as a macrophage-specific marker. Simultaneous and consecutive double-staining using Prussian blue and CD68 immunostaining were performed for co-localization of SPIONs with macrophages. Complete SPION compartmentalization by macrophages in the synovium was demonstrated by both methods, where almost all nanoparticles were located inside CD68-positive cells (Figure 4A,B and C). Conversely, a large population of macrophages did not show SPION uptake at a 7 mg Fe/ml dose. This is likely due to the SPION biodistribution, which shows a significant preference towards liver and spleen accumulation (Additional file 4) [34,35].We proceeded to characterize macrophage distribution and number in the synovium using samples from the animals that were imaged during our longitudinal MR study. Initially on day 6, macrophages in both the absence and presence of Dexa had uniformly and densely infiltrated the synovial tissue (Figure 5A,D). On day 10, a trend towards localization into subgroups at the edge of the synovium was observed in both groups; however, this pattern was more distinguishable in the Dexa group accompanied by a decrease in macrophage number (Figure 5B,E). Finally by day 13, the Dexa-treated group exhibits a unique pattern of macrophage distribution from the controls (Figure 5C,F). In the Dexa-treated group, a thick, well-defined band of macrophages was seen at the periphery of the synovium, outlining the pocket of articular edema. The location and shape of the macrophage band coincided with the elliptical line of hypointense/hyperintense signal seen on VIBE/dUTE MR images respectively. Although macrophages in the untreated group showed some localization around the edge of the synovial tissues, their distribution pattern was not as distinguishable. The analysis of the percentage of area of CD68-positive cells around the region of interest showed 6 to 7% reduction in the Dexa-treated group compared to the controls on days 10 (data not shown) and 13 (Figure 6A). The number of CD68-positive cells was also diminished at both time points (Figure 6B).As a final confirmation for both the MR and immunohistochemistry measurements, the number and area of SPION clusters were quantified. The analysis of Prussian blue staining on day 13 (Figure 7A,B) was in line with all our previous findings showing that Dexa treatment significantly reduced the area and number of SPION clusters in the synovium (Figure 7C,D).

## Discussion

Inflammatory arthritis is a chronic autoimmune disease causing recurrent episodes of joint inflammation with varying degrees of severity. These inflammatory attacks are associated with bone and cartilage erosion occurring during the first 2 years of RA onset and as early as 4 months after the first attack [36-38]. Eventually the structural damage affecting joint integrity leads to serious long-term complications including collapsed joints and permanent disability [1]. To date, commonly used diagnostic tools are limited to physical examination and scoring of certain clinical features of RA, radiographic scoring of erosion and serum markers of matrix degradation. Such techniques rely mostly on clinical signs and symptoms associated with severe inflammation and advanced joint destruction preventing early detection and intervention. Additionally, there are cases with asymptotic pathogenesis or with poor correlation between clinical symptoms and joint destruction, which present another compounding factor of RA adding to the difficulty or failure in diagnosis and treatment of the disease using those methods [2,39]. Recent clinical research has shown the advantages of using non-enhanced MRI and power Doppler ultrasound in diagnosing and assessing RA in patients [40,41]. However, both of those methods still provide no information for assessing treatment effects and disease evolution from a molecular and cellular perspective. Without a tool providing in-depth information and addressing all the limitations of the current diagnostic methods, proper RA treatment with clear remission will remain elusive.

In the present study we have assessed the effect of a well-established RA treatment Dexa, using a novel imaging technology that combines innovative MR imaging combined with nanoparticle technology, and validated these findings with supportive histochemical and immunohistochemical analyses of the imaged joint tissues. Using rats, we conducted a longitudinal evaluation of AIA progression in parallel with the cellular changes in the absence and presence of the treatment in the same animals over a period of 13 days. The T1-weighted (VIBE) and dUTE MR images of *in vivo* SPION-labeled macrophages were analyzed both qualitatively and quantitatively and the results were compared to those obtained from Gd-enhanced T1-weighted images currently used clinically for the detection of inflammation. A significant difference in the MR signal of the SPION-labeled macrophages was observed when Dexa was administered compared to the untreated controls and those differences continued over time. Immunohistochemistry and histochemistry showed cellular co-localization between macrophages and the SPIONs and we also quantified those two parameters. We found that both the number of macrophages and SPION clusters in the synovium significantly decreased with Dexa treatment.

The central role of macrophages in pathogenesis of RA is well established [11]. During inflammatory arthritis, both resident and circulating macrophages are activated and partake in the process of synovial infiltration and hyperplasia [25]. Once in the synovium, macrophages produce a multitude of pro-inflammatory cytokines and matrix degrading enzymes, which contribute to joint damage. Hence, macrophages have been the focus of many *in vitro* and *in vivo* studies and the target of RA treatments. This developed into a clinical interest in synovial macrophages where their number and response to treatment was considered a reliable biomarker for RA. Gerlag *et al*. and Haringman *et al*. assessed the response of patients to a group of RA treatments including prednisolone, methotrexate and infliximab, via the measurement of the number of CD68-positive cells in the intimal and sub-lining regions of the synovium, using knee arthroscopy before and two weeks after treatment administration [42,43]. They found that the number of sub-lining CD68-positive cells decreased in response to the treatment. Furthermore, their number was directly correlated to the 28-joint count disease activity score and standardized-response mean score used to assess the disease activity in RA patients. In contrast, using the same approach, ineffective treatment did not affect the number of sub-lining macrophages [44]. They concluded that macrophages are a sensitive and reliable biomarker that should be used to assess patients’ disease activity before and after treatment, particularly in clinical studies. The major disadvantage of their proposed technique was the use of knee arthroscopy for this type of assessment - an invasive and painful procedure.

Based on these findings we hypothesized that through *in vivo* loading and tracking of macrophages using SPIONs the effect of an RA treatment can be assessed over a period of time using MRI as a non-invasive imaging modality. Thus, we set up a treatment and imaging protocol to validate the use of SPIONs as an alternative method to assess changes in the number of macrophages *in vivo*. We chose a Dexa treatment regimen that would diminish but not entirely eliminate AIA in order to have detectable disease changes within the duration of the study. Our MR imaging protocol was focused on the VIBE sequence because it is both SPION- and Gd-sensitive and therefore, the effect of Dexa on both macrophages and synovial inflammation could be simultaneously compared. We were able to show the feasibility of *in vivo* macrophage tracking after SPION loading on MR images where a strong SPION signal was seen in the synovium of both Dexa-treated and control groups. However, this SPION signal was significantly different in its distribution between the two groups as early as 24 h after SPION injection. Whereas the Dexa-treated animals had a distinct elliptical line in the synovium, the untreated ones showed a diffuse and inhomogeneous signal. The difference between the groups continued throughout the study, where the Dexa group showed a rapid decrease in signal intensity whereas the untreated group showed some insignificant changes in that parameter and much higher variability. After verifying *in vivo* SPION labeling of macrophages using immunostaining and Prussian-blue staining, we concluded that this technique offers a consistent and reproducible elegant method to identify RA treatment and monitor cell behavior during inflammatory arthritis.

During this process we have established two methods of SPION signal quantification on MR images. The first method was qualitative scoring by an expert radiologist, which was based on identifying two parameters: SPION diffuseness and intensity, followed by blinded grading of their extent on MR images. The second method, a semi-automated three-dimensional measurement of SPION intensity and volume using dUTE-positive enhancement in all the synovium, offers the following advantages. Three-dimensional isotropic resolution allows volume analysis that avoids sampling errors when quantifying *representative* regions. As with many biological processes the edema can be an irregular shape and the SPION uptake can vary. The quantitative method used here assessed the SPION intensity as an integral over the whole synovium. An additional advantage of dUTE is the suppression of all background signals providing contrast between SPION, bone and muscle [32]. Thus, easier segmentation is therefore possible and can be automated, avoiding false positives, unlike the signal loss from bone and ligament structure on T1-weighted MRI.

In contrast, the analysis of Gd MR images, the conventionally used MR contrast agent, was less informative. Gd highlighted only synovial inflammation, which is known to decrease over time in the AIA model even in the absence of treatment. Therefore no definitive difference could be seen between the two groups. Additionally, Gd provided no information on the cellular responses to Dexa, and the lack of this critical data can be misleading in RA assessment, because it is the cellular interactions that cause most of the damage to the affected joints even in the absence of inflammatory signs such as edema. Finally, the Gd protocol required a systemic administration during each MR imaging session, which is more complicated, whereas SPIONs were injected once and tracked for up to 10 days thereafter.

As further proof of the accuracy of our findings, we quantified and correlated our MR results with histological data. Histological knee sections were immunostained using CD68 antibody as a macrophage marker and Prussian blue stained to visualize iron oxide nanoparticles. Both parameters assessed at different time points were in line with the MR data and showed a parallel decrease of both the number of macrophages and SPIONs in the joint. This evidence was the final verification that indeed our MR tracking protocol of SPION-labeled macrophages possessed the necessary sensitivity to determine minute changes in the synovial macrophage population.

Nanoparticles are a well-established tool used to label and track cells *in vitro* and *in vivo* in different disease models [45]. Namely the mononuclear phagocyte system (dendritic cells, monocytes and tissue macrophages), fibroblasts, synoviocytes, endothelial cells, tumor cells and stem cells were shown to spontaneously internalize different types of nanoparticles. In most studies, MRI was the imaging modality of choice, exploiting the superparamagnetic properties of the iron oxide nanoparticles. Due to the influx of macrophages into sites of inflammation, MRI tracking of *in vivo* and *in vitro* labeled SPIONs has become a popular method in studying conditions with macrophage involvement such as myocardial infarction, arthrosclerosis and arthritis. In cardiovascular research, the use of SPIONs has reached clinical research, where they were directly administered to patients to improve the diagnosis of arterial stenosis and atherosclerotic plaques [46-48]. In arthritis, previous studies have provided the proof of principle for the potential of *in vivo* SPION-labeled macrophages in depicting synovial hyperplasia using different animal models [13,16,19-21,49]. The outcome of this research was promising. It demonstrated the feasibility of this technology and its advantages over non-enhanced MRI and other standard contrast agents. Yet little supporting evidence has been provided by those studies on longitudinal cell-tracking and the use of the technique to assess drug effects. Beckmann *et al*. [13] evaluated the signal loss in the synovium due to SPION injection over a period of 2 weeks and compared the SPION signal in the absence and presence of Dexa. Although the objectives of both the Beckmann study and ours were similar, there are substantial differences in the design and approach used. In the Beckmann study, the animals received a new injection of SPION prior to each imaging session and continued to receive a high dose of Dexa every day, thus overlapping both SPION and treatment protocols. As SPIONs remained in the synovium between imaging sessions the results shown were a mixture of old and newly injected SPIONs. Neither qualitative nor quantitative assessment of the SPION signal was provided, only a short description of the images. Although they were able to show a difference in the number of macrophages between the two groups via histological assessment, these differences were based on measurements limited to parts of the synovium without showing any information on the effects of the SPIONs. More importantly, the repetitive SPION injection is neither a clinically feasible approach nor does it allow the tracking of the same macrophage population from the disease onset until the end of the study. It was not shown whether this large amount of SPIONs injected throughout the Beckmann study might have had any side effects on the animal and/or the evolution of the AIA model, which in turn could affect the results. In contrast, our approach was more methodical and detailed, and the animals first received the Dexa treatment followed by one dose of SPION injection. The signal loss of the SPION-labeled macrophages in the synovium was tracked over a period of time with and without Dexa, creating a protocol that is clinically applicable.

## Conclusions

In this study we have demonstrated the accuracy and robustness of MRI tracking of SPION-labeled macrophages during AIA through multiple MRI measurements and using different techniques followed by a detailed histological assessment of both the location and the number of SPIONs and macrophages for both the treated and untreated group. Our results present a novel and clinically feasible protocol to assess experimental arthritis and treatment effects that is simple, non-invasive and highly reproducible, and that can enhance preclinical drug development.

## Abbreviations

AIA: antigen induced arthritis; ANOVA: analysis of variance; B.P.: *Bordetella pertussis*; CFA: complete Freund’s adjuvant; Dexa: dexamethasone; dUTE: difference ultra-short echo time; FITC: fluorescein isothiocyanate; FOV: field of view; Gd: gadolinium chelate; ICP-OES: induced coupled plasma atomic emission spectroscopy; mBSA: methylated bovine serum albumin; MRI: magnetic resonance imaging; ON: overnight; PBS: phosphate-buffered saline; PCS: photon correlation spectroscopy apparatus; PDI: polydispersity index; PVA: poly vinyl alcohol; RA: rheumatoid arthritis; RT: room temperature; SPION: superparamagnetic iron oxide nanoparticles; T1: longitudinal relaxation time; T2: transverse relaxation time; TE: echo time; TEM: transmission electron microscopy; TI: inversion time; TR: reception time; UTE: ultra-short echo time; VIBE: three-dimensional T1 gradient echo.

## Competing interests

The authors declare they have no competing interests.

## Authors' contributions

AG prepared the manuscript, participated in the experimental study design, developed the animal model and designed the treatment protocol, participated in the MRI acquisition and qualitative scoring, supervised the histological sample preparation, analyzed and quantified the histochemical and immunohistochemical stained sections and conducted statistical analyses. LAC critically reviewed and edited the manuscript, participated in the experimental study design, optimized the MR sequences, conducted the MRI acquisition and post-acquisition processing, conducted the SPION signal quantification and statistical analyses, participated in the MRI qualitative scoring. LM was responsible for SPION development and synthesis. WW developed the custom three-dimensional volume segmentation software. FT participated in the initial pilot MRI studies for SPION detection. KG prepared the histological samples and performed the different staining. GC participated in SPION production. FE coordinated the different development steps of the custom three-dimensional volume segmentation software and critically reviewed the manuscript. MIK assisted in AIA model optimization and provided scientific advice. WBVB participated in the experimental design and animal model optimization. HH coordinated SPION production by LM and GC. JPV participated in the experimental design, critically reviewed and edited the manuscript, conducted the qualitative MRI scoring as an expert in radiology and coordinated the MRI studies. All authors have read and approved the final manuscript.

## Supplementary Material

Additional file 1 A three-dimensional magnetic resonance (MR) image stack showing the anatomy of the arthritic knee joint on day 10 post-antigen-induced arthritis (AIA) induction from the coronal and sagittal views acquired using a T1-weighted (VIBE) MRI sequence. Click here for file

Additional file 2 A three-dimensional magnetic resonance (MR) image stack showing superparamagnetic iron oxide nanoparticles (SPION) signal in relation to the anatomy of the arthritic knee joint on day 10 post-antigen-induced arthritis (AIA) induction from the coronal and sagittal views acquired using a T1-weighted (VIBE) MRI sequence. Click here for file

Additional file 3 **The evolution of antigen-induced arthritis (AIA) on magnetic resonance (MR) images in the presence and absence of dexamethasone (Dexa) without superparamagnetic iron oxide nanoparticles (SPION) administration.(A)** T1-weighted MR images of arthritic knee joints 3, 6 and 10 days post-AIA induction without any contrast enhancement. Panel (i-iii) shows representative MR images from a control (untreated) animal with AIA and panel (iv-vi) shows MR images of a Dexa-treated animal at the same timepoints. **(B)** T1-weighted MR images of arthritic knee joints 3, 6 and 10 days post-AIA induction post-gadolinium chelate (Gd) administration; images are of the same two animals shown in **A**. Gd signal is seen as a positive contrast and depicts synovial edema. Panel (i-iii) shows representative MR images from a control (untreated) animal with AIA and panel (iv-vi) shows MR images of a Dexa-treated animal at the same time points. Red arrows, quadriceps tendon to patella (dark u-shaped line); yellow arrows, patella, femur and tibia; broken orange line, edema pocket anterior to the femur. Click here for file

Additional file 4 **Whole body T1-weighted magnetic resonance (MR) images showing dose-dependent superparamagnetic iron oxide nanoparticles (SPION) accumulation in the liver.** T1-weighted MR images of a whole body scan of Lewis rats 24 h after intravenous SPION administration through the tail vein. SPION MR signal is seen as a negative contrast. Animals received no SPION **(A)**, 1 mg Fe/rat SPION **(B)** and 4 mg Fe/rat SPION **(C)** respectively. Green line outlines the liver. Click here for file
