# Supplementary figures and images for: Monitoring the effects of dexamethasone treatment by MRI using in vivo iron oxide nanoparticle-labeled macrophages
Source: Arthritis Res Ther. 2014 Jun 23;16(3):R131. doi: 10.1186/ar4588 (PMC4095600; doi:10.1186/ar4588)

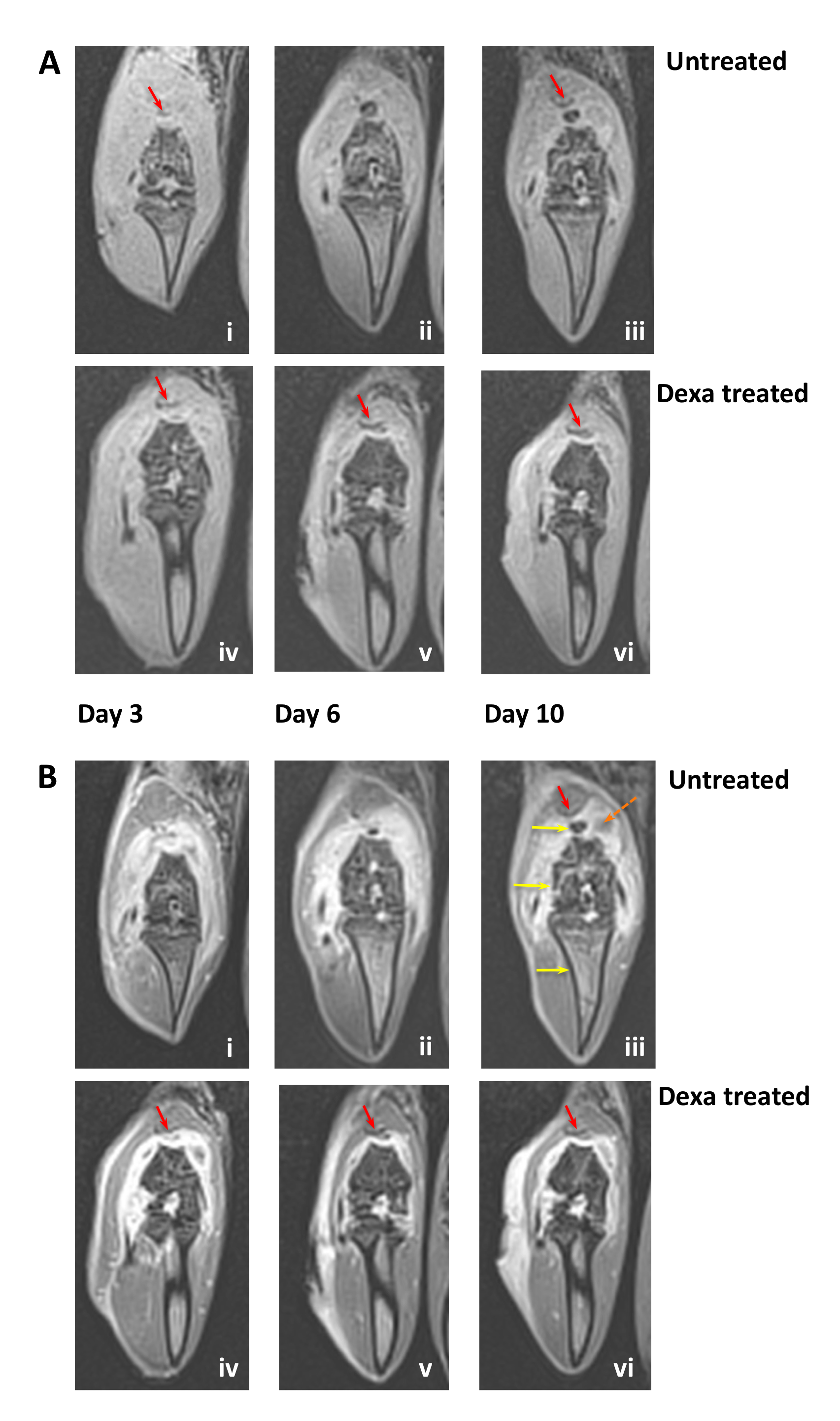

Supplement: Additional file 3 — The evolution of antigen-induced arthritis (AIA) on magnetic resonance (MR) images in the presence and absence of dexamethasone (Dexa) without superparamagnetic iron oxide nanoparticles (SPION) administration.(A) T1-weighted MR images of arthritic knee joints 3, 6 and 10 days post-AIA induction without any contrast enhancement. Panel (i-iii) shows representative MR images from a control (untreated) animal with AIA and panel (iv-vi) shows MR images of a Dexa-treated animal at the same timepoints. (B) T1-weighted MR images of arthritic knee joints 3, 6 and 10 days post-AIA induction post-gadolinium chelate (Gd) administration; images are of the same two animals shown in A. Gd signal is seen as a positive contrast and depicts synovial edema. Panel (i-iii) shows representative MR images from a control (untreated) animal with AIA and panel (iv-vi) shows MR images of a Dexa-treated animal at the same time points. Red arrows, quadriceps tendon to patella (dark u-shaped line); yellow arrows, patella, femur and tibia; broken orange line, edema pocket anterior to the femur. [file ar4588-S3.png]

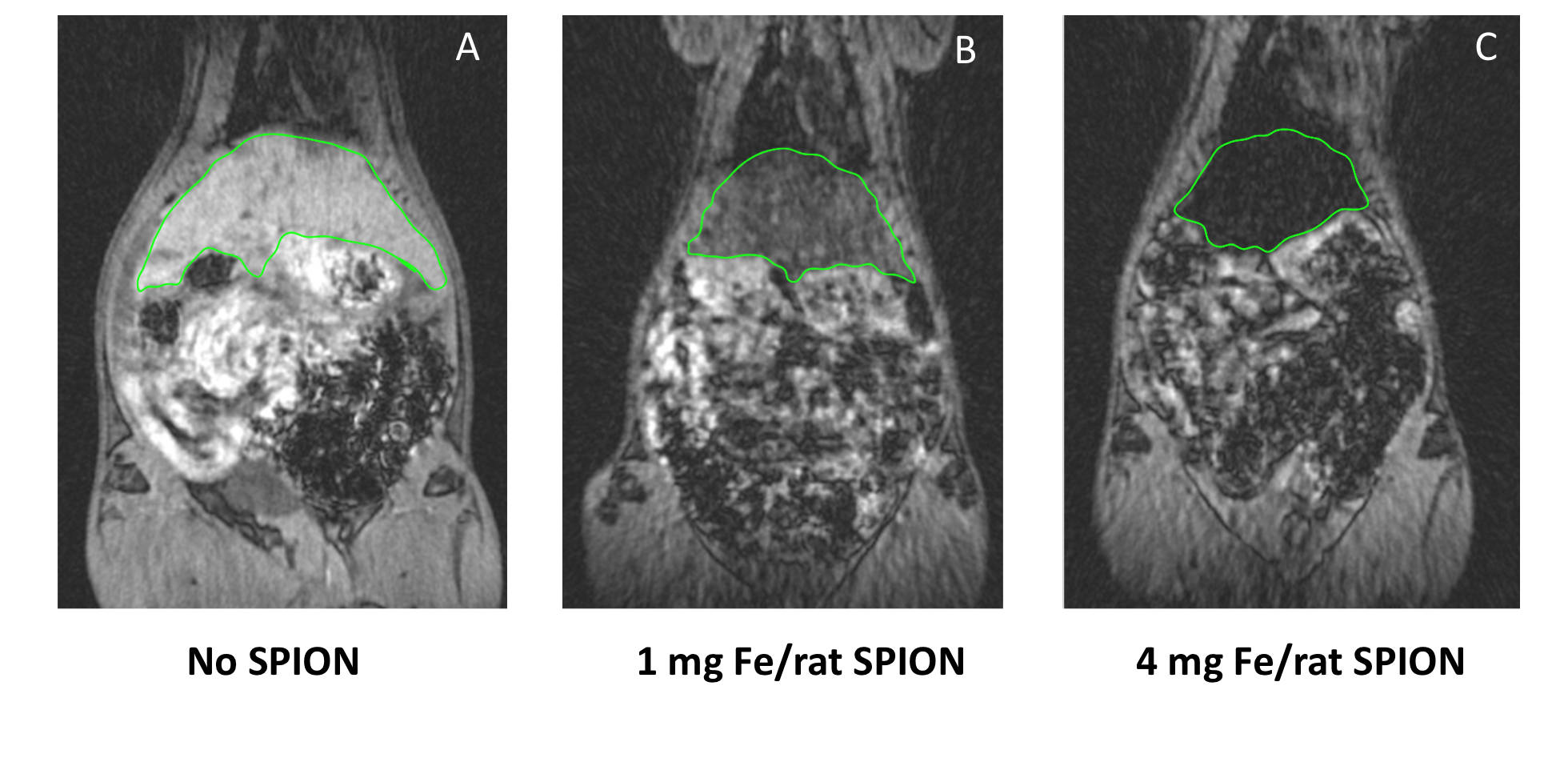

Supplement: Additional file 4 — Whole body T1-weighted magnetic resonance (MR) images showing dose-dependent superparamagnetic iron oxide nanoparticles (SPION) accumulation in the liver. T1-weighted MR images of a whole body scan of Lewis rats 24 h after intravenous SPION administration through the tail vein. SPION MR signal is seen as a negative contrast. Animals received no SPION (A), 1 mg Fe/rat SPION (B) and 4 mg Fe/rat SPION (C) respectively. Green line outlines the liver. [file ar4588-S4.png]
